# N-Carbamylglutamate Improves Production Performance and Muscle Growth by Regulating Protein Digestive Function and Muscle Protein Synthesis in Broiler Chickens

**DOI:** 10.3390/ani16101558

**Published:** 2026-05-21

**Authors:** Lingping Zhao, Shitu Tan, Wanqiao Zhang, Pei Mao, Xiaohong Wu

**Affiliations:** College of Animal Science and Technology, Henan University of Science and Technology, Luoyang 471023, China; lingpingzhao@haust.edu.cn (L.Z.); t_s_t@sina.com (S.T.); zwq1630720@163.com (W.Z.); maop17@163.com (P.M.)

**Keywords:** N-carbamylglutamate, protein digestive function, amino acids, muscle tissue, broiler chickens

## Abstract

N-carbamylglutamate (NCG) is a structural analog of N-acetylglutamate and has multifunctional roles in animal production. However, few studies have been conducted to evaluate the effects of NCG on protein digestive function and muscle growth in broiler chickens. This study investigated whether NCG could improve muscle growth via protein digestive ability and amino acid metabolism in broiler chickens. A total of 144 one-day-old male broiler chickens were randomly allocated to four treatments with six replicates of 6 broiler chickens each. The treatments were a basal diet and a basal diet with NCG supplementation at three graded levels (150 g/t, 300 g/t, and 450 g/t of NCG). The results showed that NCG supplementation significantly improved the average daily weight gain (ADG) and decreased the feed conversion ratio (FCR) compared to the control group (*p* < 0.05) and increased the weight of leg muscle and breast muscle. Furthermore, NCG supplementation significantly increased protein digestibility, the activities of amylase, trypsin and lipase, and villus height in the ileum (*p* < 0.05), which demonstrated that digestibility and absorption were improved by NCG in broiler chickens. Analysis of plasma amino acids, hormone levels and the gene expression of breast muscle revealed that NCG increased the concentration of GABA, glutamate, glutamine, leucine, threonine, valine, branched-chain amino acids and essential amino acids and the levels of testosterone and IGF-1 in plasma (*p* < 0.05). Additionally, NCG increased the expression of mTOR and P70S6K in breast muscle tissue. Therefore, NCG supplementation could be an important nutritional strategy to improve product performance, muscle growth and development, and meat yield. The 300 g/t supplementation level was found to be the practical optimum dose, as it produced effects comparable to the highest dose (450 g/t) on most parameters while offering better cost-effectiveness. This study provides new insights into the application of NCG for meat production improvement in broiler chickens.

## Simple Summary

The sufficient digestion and absorption of dietary proteins in the digestive tract have positive effects on production performance in broiler chickens. Improving protein digestibility is a strategy for increasing the yield and quality of broiler meat in poultry production. In this study, N-carbamylglutamate (NCG) supplementation significantly increased the protein digestibility, the activities of amylase, trypsin and lipase, the villus height in the ileum, and the average daily weight gain (ADG) and decreased the feed conversion ratio (FCR) and increased the weight of leg muscle and breast muscle. NCG supplementation also improved metabolism of amino acids and enhanced the levels of hormones and gene expression related to muscle protein synthesis in plasma and in breast muscle tissue, respectively. These findings demonstrate that NCG supplementation can be an important nutritional strategy to improve production performance, muscle growth and development and meat yield.

## 1. Introduction

Broiler chickens are a major source of animal protein for human consumption [1]. With a global increase in population, there is a greater demand for broiler meat. The yield and quality of broiler meat are important economic traits in poultry production and have attracted extensive research attention [2]. Low fat and high protein content are important traits for meat quality. Genetic selection for carcass yield has made great progress, especially for breast meat yield [3]. Animal nutrition is a key factor affecting meat yield and quality [4]. Therefore, feed additives are often used to improve the quality of poultry meat.

L-arginine is an essential amino acid and plays multifaceted physiological functions in animals. Previous studies have shown that Arg can regulate protein synthesis [5], improve meat quality, and enhance antioxidant function and production performance in livestock [6,7]. However, Arg is easily degraded by arginase and has a shorter half-life and can impair the absorption of dietary basic and/or structurally related amino acids including tryptophan, histidine, or lysine. Therefore, the application of Arg is limited in practical use.

N-Carbamylglutamate (NCG), a structural analog of N-acetylglutamate, has been shown to increase endogenous Arg synthesis and stimulate protein synthesis in skeletal muscle [8,9]. NCG is considered an ideal Arg alternative due to its low cost and viability in poultry and ruminant production [10,11,12].

Dietary NCG supplementation can improve reproductive performance by increasing fetal survival, litter size and birth weight in ewes and sows [13,14]. NCG can stimulate ovarian follicle development [10], improve the morphological structure of the small intestine [15], and have positive effects on eggshell quality by regulating the expression of calcium metabolism-related genes and hormone levels [16,17]. Moreover, NCG can modulate immune status and antioxidant properties in the spleen of rats [18]. In addition, NCG improved tissue development and growth performance in broilers [19]. Consequently, NCG supplementation may lead to positive outcomes for production performance in broilers. However, few studies have been conducted to evaluate the effects of NCG on digestive function and muscle growth in broiler chickens. Therefore, the objective of this study was to investigate the effects of NCG on protein digestive ability, muscle yield and amino acid metabolism in broilers. The results may provide a scientific basis for dietary supplementation of NCG to increase growth performance, meat yield and quality in broiler chickens.

## 2. Materials and Methods

### 2.1. Animal and Experimental Design

One-day-old Arbor Acres male broiler chicks were obtained from a commercial hatchery. All birds were housed in cages. The environmental temperature was maintained at approximately 34 °C in the first week post-hatching, then decreased gradually by 2–3 °C every week, dropping to 24 °C at week 4. This temperature was sustained for the remainder of the experiment. The humidity was controlled at 60% and the lighting was adjusted according to the husbandry guidelines for the Arbor Acres strain. The birds were monitored daily for health status and mortality. In a 42 d trial, a total of 144 chicks were randomly allocated to four groups with six replicate cages of 6 chicks per cage. The four experimental treatment groups included a control group (basal diet) and three treatment groups (basal diet supplemented with 150 g/t, 300 g/t, and 450 g/t of NCG). NCG (purity 97.8%) is a chemically synthesized product and was purchased from the National Feed Engineering Technology Research Center. The experimental diets were fed in 2 periods: starter (day 1–21) and finisher (day 22–42). The basal diet was formulated to meet the China Agricultural Standard nutrient recommendations [20] (The Ministry of Agriculture of the People’s Republic of China, 2004). The composition and nutrient analysis results for the basal diet are shown in Table 1. The finisher diets contained 0.4% TiO_2_ as an indigestible marker to determine nutrient digestibility. Birds were allowed to feed and drink water ad libitum, were inspected daily to ensure their welfare, and their mortality was recorded throughout the experimental period of 42 days.

### 2.2. Growth Performance

Broilers in each replicate from each treatment group were weighed to calculate the average daily gain (ADG) on day 42 of the experiment. Feed consumption was measured over the entire study period. The average daily feed intake (ADFI) and feed conversion ratio (FCR: ADFI to ADG ratio) were calculated. The weights of the leg muscle and the breast muscle were accurately recorded at the end of experiment (42 d).

### 2.3. Sample Collection

At the end of the experiment (42 d), two birds per cage (replicate) were necropsied and ileal contents were collected from the portion of the small intestine extending from the Meckel’s diverticulum to a point about 40 mm proximal to the ileocecal junction. The digesta from birds within one cage were pooled, freeze-dried (Sihuan Furui Technology Development Co., Ltd., Beijing, China), ground to pass through a 0.5 mm sieve, and stored at 4 °C for the analysis of crude protein digestibility. In addition, 12 broilers were randomly selected from each treatment and fasted for 12 h; blood samples were collected, plasma samples were obtained by centrifuging at 3000 rpm for 10 min at 4 °C and then all samples were stored at −80 °C for further analysis. The chickens were euthanized by cervical dislocation, and the carcasses were obtained. The carcasses and leg and breast meat were separated and weighed. One part of the breast muscle was collected, frozen in liquid nitrogen and stored at −80 °C until it was used for RNA extraction. Intestinal content samples were also collected using sterilized EP tubes (Nalge Nunc, Rochester, NY, USA) and stored at −80 °C until assayed. The intestine was obtained and immediately fixed in 10% neutral-buffered formalin for morphology assessment.

### 2.4. Sample Analysis

#### 2.4.1. Apparent Ileal Crude Protein Digestibility Determination

The nitrogen content of feed and ileal contents were determined using an elemental analyzer (Flash Smart Elemental Analyzer, Thermo Scientific, Bremen, Germany). The TiO_2_ concentration was analyzed via a UV-absorption spectrophotometer following a protocol outlined by Short et al. [21]. The coefficient of nitrogen digestibility was calculated using the following formula:

Digestibility (%) = {1 − [(ileal N × ileal TiO_2_)/(Feed N × Feed TiO_2_)]}

where ileal TiO_2_ was the concentration of titanium dioxide in the ileal contents, feed TiO_2_ was the concentration of titanium dioxide in the feed, and N represented the concentration of nitrogen either in the feed or the ileal contents.

#### 2.4.2. Enzyme Assays

Frozen digesta samples (100 mg) were transferred into 2 mL beating tubes. Subsequently, 1 mL of PBS was added. After homogenization by vortex mixing (1 min), the samples were centrifuged at 3500× *g* for 5 min at 4 °C. The supernatant was harvested for the analysis of digestive enzymes. The activities of digestive enzymes including amylase, protease, and lipase were determined by a microplate reader (Tecan Austria GmbH, Grödig, Austria) according to the assay kit instructions (Nanjing Jiancheng Bioengineering Institute, Nanjing, China). The enzyme activities were expressed as units per milligram or gram of protein, and the protein content of samples was determined using a commercially available bicinchoninic acid kit (Nanjing Jiancheng Bioengineering Institute).

#### 2.4.3. Histological Observation

Ileum samples fixed in 4% phosphate-buffered formaldehyde solution were dehydrated and then embedded in paraffin. Serial sections were cut at approximately 5 µm, deparaffinized in xylene, rehydrated, and stained with hematoxylin and eosin as previously described [22]. Sections were observed with light microscopy at 10 × 10 magnification. The villus height (from the tip to the villus–crypt junction) and crypt depth (the depth between adjacent villi) were measured. The villus height-to-crypt depth ratio was calculated from these measurements.

#### 2.4.4. Amino Acid Determination

Analysis of the free amino acids in the plasma was performed using ultra-high-performance liquid chromatography coupled with mass spectrometry (UHPLC-MS/MS, Thermo Fisher, Waltham, MA, USA) and they were quantified by isotope internal standard. Mobile phase A was 5 mM ammonium acetate in water and mobile phase B was acetonitrile. The column temperature was set to 45 °C. The autosampler temperature was set to 4 °C, and the injection volume was 2 μL.

The MS conditions were as follows: spray voltage = 3.3 kV, sheath gas = 40 Arb, auxiliary gas = 10 Arb, sweep gas = 1 Arb, ion transfer tube temperature = 325 °C, vaporizer temperature = 350 °C.

#### 2.4.5. Hormone Assays

The levels of testosterone and insulin-like growth factor 1 (IGF-1) in the plasma were determined using commercially available kits (Nanjing Jiancheng Bioengineering Institute).

#### 2.4.6. Messenger RNA Quantification

Total RNA was extracted from leg muscle using TRIzol isolation reagent (TaKaRa Bio Inc., Otsu City, Japan) following the manufacturer’s protocol. Real-time PCR was performed on a Bio-Rad CFX96 Detection System (Bio-Rad Laboratories, Inc., Singapore) using SYBR Green PCR Master Mix (Takara, Dalian, China). The sequences of PCR primers for the target genes are shown in Table 2, and the primer sequences were designed and synthesized by Takara Bio Inc.

Each real-time PCR was performed in triplicate and normalized with the housekeeping gene β-actin. Relative gene expression data were determined using the 2^−ΔΔCt^ method [23].

### 2.5. Statistical Analysis

All data were expressed as mean ± standard deviation (SD). One-way analysis of variance (ANOVA) was used to determine whether significant differences existed among the treatment groups. All parameters were compared using Tukey’s test. *p*-values < 0.05 and *p* ≤ 0.01 were considered statistically significant and highly significant differences, respectively. The statistical analyses were performed using SPSS Statistics version 22.0.

## 3. Results

### 3.1. Growth Performance and Weight of Leg Muscle and Breast Muscle

Table 3 shows the growth performance and weight of the leg muscle and breast muscle of broiler chickens fed different experimental diets for 42 d. At the end of the experiment, ADG in the broilers was significantly higher in the three NCG treatment groups compared with the control group (*p* < 0.05), while ADG did not differ significantly among the three NCG treatment groups (*p* > 0.05). There were no significant differences in ADFI among groups (*p* > 0.05). Diets supplemented with NCG at 300 g/t and 450 g/t resulted in lower FCR compared with the control group or the 150 g/t NCG group (*p* < 0.05). The weight of the leg muscle and breast muscle significantly increased in the NCG treatment groups, and the highest leg muscle weight was recorded in the 450 g/t NCG group (Table 3).

### 3.2. Apparent Ileal Crude Protein Digestibility

**Figure 1:**
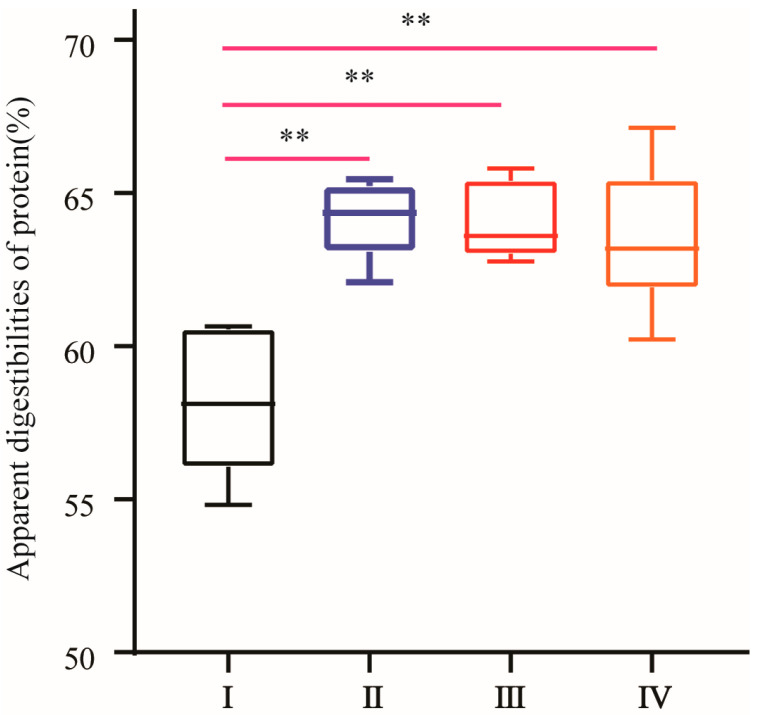
Effect of NCG on apparent ileal crude protein digestibility. (I) Control group, (II) 150 g/t of NCG treatment group, (III) 300 g/t of NCG treatment group, (IV) 450 g/t of NCG treatment group. ** means the difference between the different groups is significant at the 0.01 level.

The results showed that the digestibility coefficient of the apparent ileal crude protein was higher in broilers fed one of the three NCG treatments than in broilers receiving no NCG supplementation (*p* < 0.01). No differences in protein digestibility were found among the three NCG treatment groups. These results were presented in Figure 1.

### 3.3. Enzyme Activity

**Figure 2:**
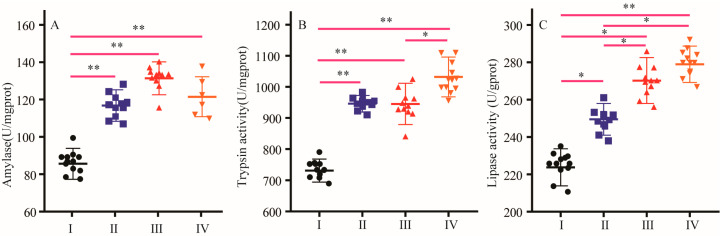
Effect of NCG on activities of amylase, trypsin, lipase. (**A**) Amylase activity, (**B**) trypsin activity, (**C**) lipase activity; error bars represent SD. (I) Control group, (II) 150 g/t of NCG treatment group, (III) 300 g/t of NCG treatment group, (IV) 450 g/t of NCG treatment group. * or ** means the difference between the different groups is significant at the 0.05 or 0.01 level.

As shown in Figure 2, the activities of amylase, trypsin, and lipase were significantly higher in the three NCG treatment groups than in the control group. Trypsin and lipase activities were substantially and dose-dependently increased by NCG (Figure 2B,C).

### 3.4. Histological Changes in Ileum

**Figure 3:**
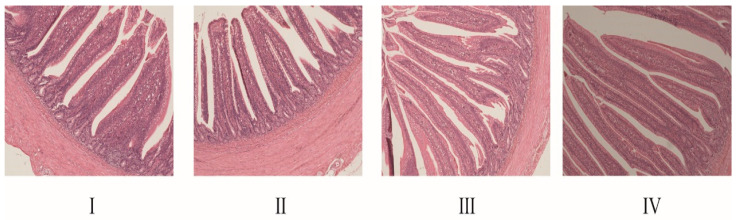
Effect of NCG on morphology of ileum in broiler chickens. (**I**) Control group, (**II**) 150 g/t of NCG treatment group, (**III**) 300 g/t of NCG treatment group, (**IV**) 450 g/t of NCG treatment group. All histological images are at 10 × 10 magnification.

The results for the ileum morphological parameters, including the villus height, crypt depth and the ratio of villus height to crypt depth, are shown in Table 4 and Figure 3. The villus height in the ileum was larger in the 300 g/t and 450 g/t NCG groups when compared to the 0 and 150 g/t NCG groups (*p* < 0.05). Crypt depth and the villus height-to-crypt depth ratio did not differ among groups (*p* > 0.05).

### 3.5. Amino Acid Concentrations

Supplementation of feed with NCG increased the concentration of gamma-aminobutyric acid (GABA), glutamate, glutamine, leucine, threonine, valine, branched-chain amino acids (BCAAs), and essential amino acids (EAAs) in plasma compared with the control group (no supplementation), but no dose effect was observed. The results were statistically significant, as indicated in Table 5.

### 3.6. Plasma Hormone Levels

**Figure 4:**
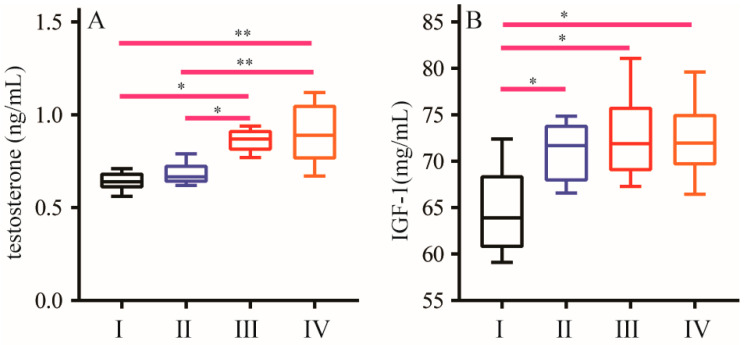
Effect of NCG on plasma hormone levels. (**A**) Plasma testosterone levels. (**B**) Plasma IGF-1 levels. (I) Normal group; (II) 150 g/t of NCG treatment group; (III) 300 g/t of NCG treatment group; (IV) 450 g/t of NCG treatment group. * or ** means the difference between the different groups is significant at the 0.05 or 0.01 level.

The levels of testosterone and IGF-1 in plasma are shown in Figure 4. Compared with the control group, the 300 g/t and 450 g/t NCG treatment groups had higher testosterone levels (*p* < 0.05), while the 150 g/t NCG treatment group did not differ significantly from the control group. All three NCG treatment groups had higher IGF-1 levels than the control group (*p* < 0.05), but the differences among the NCG treatment groups were not significant.

### 3.7. Gene Expression

**Figure 5:**
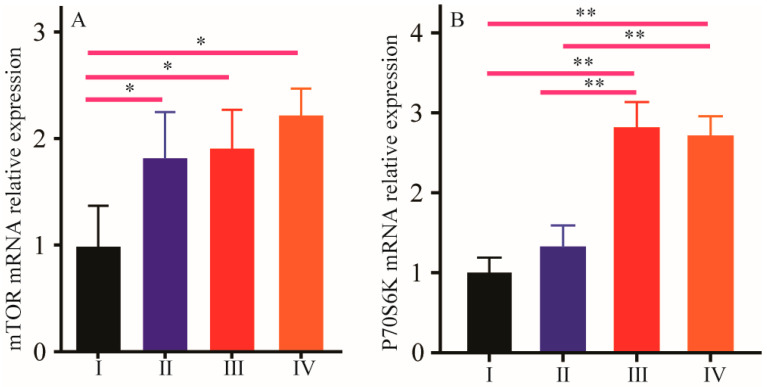
Effect of NCG on gene expression. (**A**) mTOR mRNA; (**B**) P70S6K mRNA. (I) Normal group; (II) 150 g/t of NCG treatment group; (III) 300 g/t of NCG treatment group; (IV) 450 g/t of NCG treatment group. * or ** means the difference between the different groups is significant at the 0.05 or 0.01 level.

The effects of NCG on the expression of the mammalian target of rapamycin (mTOR) and P70 ribosomal protein S6 kinase (P70S6K) genes were evaluated in the breast muscle of broiler chickens. The results are shown in Figure 5. We observed that NCG treatment increased the expression of mTOR (*p* < 0.05) compared with the control group, with no dose effect observed. The expression of P70S6K was not affected by the 150 g/t NCG treatment compared with the control group; however, the 300 g/t and 450 g/t NCG treatments resulted in higher expression of P70S6K than the 150 g/t NCG treatment or the control group (*p* < 0.01).

## 4. Discussion

The effects of NCG on the protein digestibility, intestinal histomorphology, amino acid metabolism, and muscle protein synthesis ability of broiler chickens are still not well-documented. In this study, we found that NCG supplementation significantly improved protein digestibility in broiler chickens. Proteolytic enzyme activity is required to break down dietary protein into a form ready for digestion and absorption [24]. Subsequently, trypsin activity and the anatomy of the digestive tract were tested, and the results showed that NCG supplementation increased trypsin activity and villus height. In addition, our results found that the activities of amylase and lipase were also increased by NCG intake in broiler chickens. These changes likely contributed to the improvement of production performance. We subsequently evaluated production performance in broiler chickens, and the results showed that NCG increased the ADG, improved the FCR, and increased the weight of leg muscle and breast muscle. Therefore, these results indicated that NCG exerted a growth-promoting effect by facilitating the digestion and absorption of nutrients and improving the muscle meat yield in broiler chickens.

Chicken meat is an excellent source of animal protein for human nutrition [25]. Petracci et al. reported that breast meat yield exceeds one-fifth of the weight of the bird [3]. A high yield of breast and leg meat is expected in broiler chicken production because they are the main sites of muscle protein accretion.

In broiler chickens, the rapid growth of breast muscle and leg muscle requires sufficient amino acids (AAs) for skeletal muscle protein synthesis. Therefore, dietary protein digestion, AA absorption, and plasma amino acid availability are very important. Muscle protein synthesis is driven by dietary protein digestion, plasma amino acid availability and intramuscular signaling [26,27,28]. In the present study, the contents of free blood amino acids including arginine, GABA, glutamate, glutamine, leucine, threonine and valine were significantly increased by NCG supplementation; specifically, the BCAA and EAA content. It has been suggested that specific amino acids such as leucine, isoleucine and arginine could promote anabolic responses [29,30] and deficiencies of lysine and methionine could attenuate muscle protein synthesis [31]. It has been demonstrated that arginine is essential in catabolic conditions, such as pregnancy, infancy and trauma [32]. Arginine can also improve the synthesis of protein and milk yield [33]. Together with the present study, we propose that the increase in leg muscle and breast muscle mass following NCG supplementation is closely associated with increased synthesis and metabolism of endogenous arginine [34].

Additionally, BCAAs play a crucial role in tissue maintenance and growth by stimulating protein synthesis in broiler chickens [35]; some EAAs have a greater influence on the growth of chick muscles [36], and a higher content of EAAs can improve the nutritional value and quality of chicken meat [25]. These studies indicated that higher protein digestion increases the blood amino acid content, which not only provides enough building blocks for muscle protein synthesis, but can also stimulate muscle protein synthesis.

It is widely recognized that muscle mass is regulated by a variety of hormones such as testosterone, IGF-1 and GH [37,38,39]. Previous studies have suggested that testosterone could influence muscle metabolism and growth [40], and exogenous testosterone supplementation improved breast muscle performance [41]. IGF-1 can stimulate the growth of skeletal muscle by enhancing the rate of protein synthesis [42], and the lower serum IGF-1 levels in chickens are associated with lower breast muscle mass and yield [43]. Reminiscent of previous reports, we found that NCG supplementation resulted in higher levels of testosterone and IGF-1 compared with the control group [44], suggesting that NCG could regulate muscle mass and this change is associated with enhanced testosterone and IGF-1 levels in serum. Proposed mechanisms for increasing muscle mass include enhanced synthesis of muscle-fiber proteins, reduced protein degradation and activation of the mammalian target of rapamycin (mTOR) signaling pathway [45,46]. Several studies have showed that BCAAs, glycine and glutamine can activate the mTOR pathway or increase muscle growth [47,48,49]. Ribosomal protein kinase S6 (P70S6K) is a downstream positive regulator of mTOR. Loss of muscle mass is associated with decreased expression of the P70S6K gene [50]. In the present study, NCG supplementation increased the expression of the mTOR and P70S6K genes in the breast muscle of broiler chickens. It is possible that NCG supplementation could improve muscle mass through activation of the mTOR/P70S6K signaling pathway. Other hormones and genes such as GH, cortisol, and PI3K/AKT are closely involved in muscle growth regulation and thus more investigation is needed to provide a further scientific basis for the application of NCG.

Based on the results of the present study, the improvement in production performance following NCG supplementation may result from the combined effects of factors such as amino acids, hormone levels, and gene expression. Notably, the supplementation of 300 g of NCG per ton of basal diet achieved desirable results. A supplemental level of 300 g NCG per ton may be the practical optimum dose based on the present study because higher supplemental level of NCG did not consistently improve responses and would result in higher costs.

## 5. Conclusions

In conclusion, this study demonstrated that supplementation with medium or high doses of NCG (300 g/t or 450 g/t) enhanced the growth performance and weight of leg muscle and breast muscle, improved the protein digestibility, ileal morphological parameters, and activities of amylase, trypsin, and lipase, and increased the concentrations of arginine, GABA, glutamate, glutamine, leucine, threonine, and valine, and particularly the concentration of BCAAs and EAAs, in broiler chickens. In addition, NCG supplementation (300 g/t or 450 g/t) significantly increased serum testosterone and IGF-1 levels and the expression of mTOR and P70S6K genes in the breast muscle of broiler chickens. These findings suggest that NCG has positive effects on muscle growth and development in broiler chickens and may represent an important strategy to improve meat production. The 300 g/t supplementation level was found to be the practical optimum dose, as it produced effects comparable to the highest dose (450 g/t) for most parameters while offering better cost-effectiveness. Future studies are needed to clarify the mechanism of action of NCG on muscle mass improvement and meat quality.

## Figures and Tables

**Table 1:** Ingredient composition and nutritional composition of basal diets.

Ingredient and Composition	Day 1 to 21: Starter (%)	Day 22 to 42: Finisher (%)
Corn	55.13	57.70
Soybean meal	38	36
Soybean oil	3.0	3.0
Limestone	1	1
Dicalcium phosphate	1.7	1.2
NaCl	0.3	0.3
Lys	0.25	0.3
Met	0.25	0.16
Trace mineral premix ^1^	0.1	0.1
Vitamin–mineral premix ^2^	0.14	0.14
Choline chloride	0.13	0.10
Total	100	100
Calculated nutrient (%)		
Crude protein	21.09	20.03
Ca	1.05	0.85
P	0.56	0.45
Available P	0.42	0.38
Lys	1.22	1.04
Met	0.52	0.48
AME (kcal/kg)	3050	3010

^1^ Trace mineral premix provided the following per kg of diet: manganese, 88 mg; zinc, 95 mg; iron, 100 mg; copper, 12.5 mg; selenium, 0.3 mg; and iodine, 0.7 mg. ^2^ Vitamin premix provided the following per kg of diet: vitamin A, 8000 IU; vitamin D3, 2000 IU; vitamin E, 30 IU; vitamin K, 2.0 mg; thiamine, 2.0 mg; riboflavin, 5.0 mg; niacin, 0.15 mg; vitamin B6, 4.0 mg; vitamin B12, 0.02 mg; pantothenic acid, 40.0 mg; folic acid, 10.0 mg; and biotin, 1.0 mg.

**Table 2:** Primer sequences for quantitative real-time PCR.

Gene	Forward Primer (5′−3′)	Reverse Primer (5′−3′)	Fragment Size (bp)	GenBank Accession Number
mTOR	AGCTCACACCCCTGTTTGAA	GCAACGTGCTCTCACAATG	121	XM_417614.6
P70S6K	AAGTTGAATAGGAGGGC	GAAGATGTCACTGCGAAT	148	NM_205816.1
β-actin	ATCCGGACCCTCCATTGTC	AGCCATGCCAATCTCGTCTT	120	NM205518

**Table 3:** Effects of NCG on growth performance and weight of leg muscle and breast muscle.

Item	Levels of NCG			
	0	150 g/t	300 g/t	450 g/t
ADG (g/day)	55.29 ± 2.03 ^b^	57.68 ± 3.96 ^a^	58.22 ± 4.61 ^a^	59.13 ± 3.98 ^a^
ADFI (g)	89.44 ± 6.45	91.51 ± 8.17	89.96 ± 7.86	88.63 ± 8.19
FCR	1.61 ± 0.08 ^a^	1.60 ± 0.10 ^a^	1.53 ± 0.06 ^b^	1.50 ± 0.08 ^b^
Carcass weight (g)	12,028 ± 72	1349 ± 61	1335 ± 85	1436 ± 74
Weight of leg muscle (g)	456.8 ± 41.8 ^c^	516.9 ± 45.6 ^b^	505.6 ± 49.1 ^b^	571.5 ± 46.1 ^a^
Weight of breast muscle (g)	310.6 ± 42.7 ^b^	345.4 ± 55.7 ^a^	347.1 ± 46.5 ^a^	346.2 ± 43.9 ^a^

Data are expressed as mean ± SD. In the same row, values with the same lowercase letters or without letters are not significantly different (*p* > 0.05), while with different lowercase letters are significantly different (*p* < 0.05).

**Table 4:** Effects of NCG on ileum morphology in broilers.

Item	Levels of NCG			
	0	150 g/t	300 g/t	450 g/t
Villus height (μm)	879.1 ± 53.2 ^b^	893.6 ± 36.5 ^b^	968.3 ± 28.9 ^a^	947.2 ± 40.1 ^a^
Crypt depth (μm)	112.6 ± 13.1	106.5 ± 14.4	117.4 ± 11.5	105.9 ± 15.4
Villus height/crypt depth	7.85 ± 0.09	8.52 ± 0.12	8.27 ± 0.07	8.93 ± 0.08

Villus height and crypt depth were measured from 10 villi per bird and averaged, and only complete, vertically oriented villi were measured. Data are expressed as mean ± SD. In the same row, values with the same lowercase letters or without letters are not significantly different (*p* > 0.05), while with different lowercase letters are significantly different (*p* < 0.05).

**Table 5:** Plasma concentrations of 22 amino acids (μmol/L).

Amino Acids	Levels of NCG			
	0	150 g/t	300 g/t	450 g/t
Alanine	390.5 ± 15.0	410.3 ± 8.4	395.2 ± 12.9	407.9 ± 11.54
Arginine	174.3 ± 15.8 ^b^	167.4 ± 12.1 ^b^	186.6 ± 13.9 ^a^	191.6 ± 14.2 ^a^
Asparagine	36.16 ± 3.34	40.53 ± 2.84	38.21 ± 4.73	36.42 ± 8.07
Aspartic acid	27.18 ± 2.15	27.76 ± 2.39	27.35 ± 3.06	28.94 ± 2.97
Cysteine	39.19 ± 4.56	38.78 ± 4.40	37.78 ± 3.55	36.22 ± 2.58
GABA	0.91 ± 0.19 ^b^	1.57 ± 0.28 ^a^	1.44 ± 0.13 ^a^	1.52 ± 0.17 ^a^
Glutamate	1589.4 ± 251.9 ^b^	1704.6 ± 431.7 ^a^	1699.0 ± 333.5 ^a^	1709.9 ± 379.8 ^a^
Glutamine	486.7 ± 32.7 ^b^	542.8 ± 25.6 ^a^	585.7 ± 24.7 ^a^	599.0 ± 27.8 ^a^
Glycine	207.3 ± 15.6	205.3 ± 11.1	211.6 ± 10.4	215.9 ± 15.9
Histidine	97.89 ± 8.82	97.60 ± 7.49	95.14 ± 9.62	96.33 ± 8.13
Isoleucine	72.09 ± 7.26	69.68 ± 14.11	65.27 ± 13.26	67.51 ± 12.03
Leucine	176.4 ± 22.0 ^b^	192.7 ± 19.4 ^a^	218.9 ± 17.6 ^a^	209.7 ± 15.8 ^a^
Lysine	229.2 ± 48.4	241.1 ± 25.5	238.9 ± 36.4	235.6 ± 29.1
Methionine	59.45 ± 2.74	62.64 ± 3.89	57.45 ± 4.33	61.40 ± 5.98
Phenylalanine	85.28 ± 2.05	83.47 ± 5.23	84.49 ± 4.77	83.55 ± 4.12
Proline	218.9 ± 9.6	232.5 ± 12.3	249.1 ± 11.0	244.0 ± 9.1
Serine	483.3 ± 31.4	483.2 ± 45.9	498.4 ± 35.6	477.7 ± 31.6
Taurine	22.85 ± 1.51	25.51 ± 2.19	24.35 ± 2.58	25.09 ± 3.17
Threonine	179.1 ± 13.4 ^b^	205.1 ± 28.4 ^a^	219.5 ± 19.3 ^a^	212.5 ± 15.1 ^a^
Tryptophan	42.75 ± 5.19	39.44 ± 5.68	44.78 ± 2.97	41.09 ± 3.64
Tyrosine	38.54 ± 1.67	33.07 ± 2.51	36.59 ± 5.03	37.65 ± 4.77
Valine	160.3 ± 9.3 ^b^	171.3 ± 8.9 ^a^	179.1 ± 7.5 ^a^	176.9 ± 9.2 ^a^
BCAA	411.5 ± 38.9 ^b^	432.9 ± 29.9 ^b^	462.7 ± 30.5 ^a^	453.5 ± 27.7 ^a^
EAA	1006 ± 64 ^b^	1063 ± 63 ^b^	1105 ± 59 ^a^	1086 ± 55 ^a^
Total AA	4979 ± 634	5068 ± 595	5185 ± 659	5184 ± 547

GABA = Gamma-aminobutyric acid; BCAAs = branched-chain amino acids; EAA = essential amino acid; AAs = amino acids. Data are expressed as mean ± SD. In the same row, values with the same lowercase letters or without letters are not significantly different (*p* > 0.05), while values with different lowercase letters are significantly different (*p* < 0.05).

## Data Availability

The original contributions presented in this study are included in the article. Further inquiries can be directed to the corresponding authors.

## Abbreviations

The following abbreviations are used in this manuscript:

NCG	N-carbamylglutamate
ADG	Average daily weight gain
ADFI	Average daily feed intake
FCR	Feed conversion ratio
GABA	Gamma-aminobutyric acid
IGF-1	Insulin-like growth factor 1
mTOR	Mammalian target of rapamycin
P70S6K	P70 ribosomal protein S6 kinase
